# Reconstructive Strategies After Mastectomy: Comparative Outcomes, PMRT Effects, and Emerging Innovations

**DOI:** 10.3390/jcm15010147

**Published:** 2025-12-24

**Authors:** Mihai Stana, Nicoleta Aurelia Sanda, Marius Razvan Ristea, Ion Bordeianu, Adrian Costache, Florin Teodor Georgescu

**Affiliations:** 1 General Surgery Department, Bucharest Emergency Clinical Hospital, 014461 Bucharest, Romania; dr.stanamihai@yahoo.com (M.S.); florin.georgescu1@yahoo.com (F.T.G.); 2 General Surgery Department, Carol Davila University of Medicine and Pharmacy, 050474 Bucharest, Romania; 3 Plastic Surgery Department, Carol Davila University of Medicine and Pharmacy, 050474 Bucharest, Romania; ristea.marius1@yahoo.com; 4 Plastic Surgery Department, Faculty of Medicine, Ovidius University, 900470 Constanta, Romania; ion_bordeianu@hotmail.com; 5 Pathology Department, Carol Davila University of Medicine and Pharmacy, 050474 Bucharest, Romania

**Keywords:** acellular dermal matrix, autologous breast reconstruction, breast cancer, BREAST-Q, complications, implant-based reconstruction, innovations, postmastectomy radiotherapy (PMRT), robotic surgery, sensory nerve coaptation

## Abstract

**Background:** Advances in breast reconstruction have transformed the recovery pathway for women undergoing mastectomy. What was once viewed mainly as a cosmetic option is now recognized as part of modern oncologic care, restoring not only body image but also confidence and quality of life. Yet, surgeons still face the same central dilemma: choosing between implant-based (IBR) and autologous reconstruction (ABR), particularly when postmastectomy radiotherapy (PMRT) is planned. **Methods:** We reviewed major studies published between 2014 and 2024, combining evidence from observational cohorts and recent meta-analyses that together report on more than 60,000 reconstructed breasts. Outcomes of interest included surgical complications, reconstructive failure, BREAST-Q patient-reported domains, and the impact of PMRT on both techniques. Data were interpreted in light of contemporary reconstructive innovations such as prepectoral implants, acellular dermal matrices, and robotic or sensory-nerve–enhanced autologous procedures. **Results:** Autologous reconstruction generally provided higher satisfaction and better psychosocial and sexual well-being, particularly in patients who received PMRT. Implant-based reconstruction offered faster recovery and shorter hospitalization but was more vulnerable to capsular contracture and reconstructive loss after irradiation. Across all eligible cohorts, reconstruction—immediate or delayed—did not increase local recurrence or compromise overall survival when adjuvant therapy was delivered without delay. **Conclusions:** Both IBR and ABR are oncologically safe and contribute meaningfully to recovery after mastectomy. Future progress will depend on combining precise surgical execution with new technologies—prepectoral implant positioning, robotic flap harvest, and sensory nerve coaptation—to achieve durable, natural, and patient-centered reconstruction.

## 1. Introduction

Breast reconstruction has become a defining element of modern breast cancer care. For many women, it represents more than the physical replacement of a breast—it restores a sense of identity, confidence, and normalcy after the trauma of mastectomy [1,2]. Improvements in systemic therapy and oncologic surgery have steadily increased survival, and as a result, the focus of treatment has shifted from survival alone to the quality of life that follows [3,4,5].

Surgeons today must navigate a complex balance: achieving aesthetic and functional restoration while maintaining oncologic safety and ensuring that adjuvant therapy is not delayed [6,7]. The choice between implant-based reconstruction (IBR) and autologous reconstruction (ABR) often hinges on factors such as anticipated radiotherapy, patient comorbidities, and personal preference. Postmastectomy radiotherapy (PMRT) improves local control and survival but is known to affect reconstructive outcomes, especially in implant-based settings [8,9]. Autologous reconstruction tends to withstand radiation better and offers more natural results, but at the cost of longer surgery and donor-site recovery [10]. At the same time, patient expectations have evolved—outcomes are now measured not only in complication rates but also through validated patient-reported tools like the BREAST-Q, which capture psychosocial and sexual well-being as essential parts of recovery [11,12].

The absence of reconstruction after mastectomy has been linked to persistent impairments in body image, reduced social confidence, and lower satisfaction with the overall treatment experience [1,6]. These effects often extend well beyond the initial postoperative period and reflect the broader psychosocial impact that loss of the breast mound can impose on daily life. Recognizing these implications reinforces the importance of offering balanced, evidence-based counseling on reconstructive options as part of modern, patient-centered breast cancer care.

Still, access to reconstructive care remains far from equal. Even within Europe, large regional differences persist in reconstruction rates, referral timing, and the availability of specialized teams [13]. Similar inequalities have been documented in Central and Eastern Europe, where late-stage presentation and limited reconstructive infrastructure still affect long-term outcomes [14]. Recognizing these disparities underscores the importance of contextualizing surgical results within the broader framework of healthcare accessibility and patient experience.

In recent years, the field has entered a new phase—one defined by innovation and integration. The shift toward prepectoral implant placement supported by acellular dermal matrices (ADM) has reduced postoperative pain and animation deformity [15,16]. Hybrid techniques that combine implants with fat grafting are improving contour and radiation tolerance [17]. Robotic and endoscopic flap harvests are minimizing donor-site morbidity [18], while sensory nerve coaptation is beginning to restore not just shape, but sensation and emotional completeness [19,20].

The present review brings together comparative data from recent observational cohorts and meta-analyses to examine clinical, oncologic, and patient-reported outcomes in implant-based and autologous reconstruction. Particular attention is given to the effects of PMRT and to the emerging innovations that are reshaping the future of reconstructive oncology.

## 2. Methods

### 2.1. Search Strategy and Study Selection

A systematic search was conducted in the PubMed/MEDLINE and Embase databases to identify relevant studies published between January 2014 and December 2024, following the PRISMA 2020 recommendations. The search strategy combined controlled vocabulary and free-text terms related to breast reconstruction, mastectomy, implant-based reconstruction, autologous reconstruction, postmastectomy radiotherapy (PMRT), complications, and patient-reported outcomes (BREAST-Q). Reference lists of eligible studies and major systematic reviews were also hand-searched to identify additional relevant publications.

**Figure 1:**
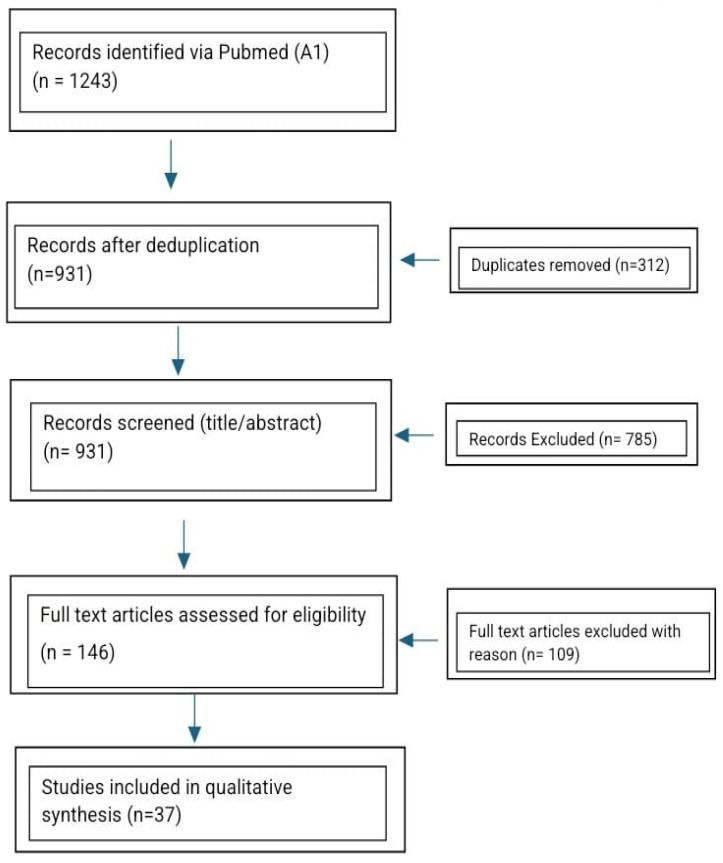
PRISMA 2020 flow diagram of study selection.

The literature search was performed in PubMed, MEDLINE, Scopus, and Web of Science using predefined keywords: “breast reconstruction”, “implant-based reconstruction”, “autologous reconstruction”, “DIEP flap”, “postmastectomy radiotherapy”, “prepectoral reconstruction”, and “patient-reported outcomes”. Search filters were restricted to human studies published in English between January 2014 and October 2024. Two independent reviewers (N.S. and R.V.C.) screened all titles and abstracts, followed by full-text evaluation of potentially eligible studies. Any disagreements regarding inclusion were resolved by discussion and consensus. We included comparative clinical studies, prospective or retrospective cohorts, randomized controlled trials, and systematic reviews or meta-analyses that reported outcomes for implant-based and/or autologous breast reconstruction after mastectomy, with or without postmastectomy radiotherapy. Exclusion criteria comprised non-comparative studies, case reports, small case series (<30 patients), editorials, expert opinions, conference abstracts, technical notes, and non-English publications. Study selection followed PRISMA guidelines, and the screening process is illustrated in the updated PRISMA 2020 flow diagram (Figure 1).

All retrieved records were imported into Zotero reference management software (Corporation for Digital Scholarship, Vienna, VA, USA; version 6.0), and duplicates were removed automatically. Two reviewers independently screened titles and abstracts to determine potential eligibility. Full-text articles meeting the preliminary inclusion criteria were subsequently reviewed in detail to confirm inclusion. Discrepancies were resolved by consensus or, if needed, through consultation with a senior reviewer.

The identification, screening, eligibility, and inclusion process is summarized in Figure 1 (PRISMA 2020 flow diagram), which outlines the number of records retrieved, screened, excluded, and ultimately included in the final synthesis. The detailed search strings and exclusion log are provided in Appendix A.

PRISMA 2020 flow diagram illustrating study selection (n = 37 included studies).

Records identified via PubMed and Embase (n = 1243) were screened after deduplication (n = 931). After title and abstract screening, 146 full-text articles were assessed for eligibility, and 37 met the inclusion criteria. The diagram summarizes the identification, screening, eligibility, and inclusion stages of the review, following the PRISMA 2020 guidelines. Complete search strings and the exclusion log are provided in Appendix A.

### 2.2. Eligibility Criteria

Studies were eligible if they met the following conditions: published between 2014 and 2024; reported surgical outcomes, complications, aesthetic results, or patient-reported outcomes after breast reconstruction; included adult patients (≥18 years), regardless of sex; and were published in English and indexed in MEDLINE. Accepted study designs comprised randomized controlled trials (RCTs), prospective and retrospective cohort studies, case–control studies, systematic reviews, and meta-analyses.

Exclusion criteria included non-English language, preclinical or animal research, pediatric series, and case reports with fewer than five patients. Editorials, letters, and commentaries without primary data, as well as conference abstracts lacking full-text publication, were also excluded.

### 2.3. Data Extraction and Outcomes

For each eligible study, the following information was extracted: first author, year of publication, country, study design, sample size, patient demographics, reconstructive technique (implant-based or autologous, timing, and plane of placement), exposure to adjuvant therapy, outcomes evaluated (complications, reoperations, patient-reported outcomes), duration of follow-up, and key findings. Data extraction was performed independently by two reviewers using a standardized template.

The primary outcomes were surgical complications and reconstructive failure, whereas secondary outcomes included BREAST-Q domains (satisfaction, psychosocial and sexual well-being), unplanned reoperation, readmission, and oncologic endpoints such as local recurrence, disease-free survival, and overall survival.

Table 1 summarizes the database search terms and inclusion filters applied across all eligible studies.

An overview of all studies included in this review—covering publication years, study design, reconstructive technique, sample size, and reported outcomes—is provided in Table 1, while Table 2 presents representative comparative cohorts detailing design, patient population, exposure to radiotherapy, follow-up period, and primary outcomes.

### 2.4. Quality Assessment

The methodological quality of the included studies was appraised using validated instruments appropriate to the study design. The Cochrane Risk of Bias 2 (RoB 2) tool was applied to randomized trials, the Newcastle–Ottawa Scale (NOS) to observational studies, and the AMSTAR-2 checklist to systematic reviews and meta-analyses. Each study was assessed independently by two reviewers, with disagreements resolved by consensus.

Because of heterogeneity in reporting, graphical bias summaries were not included in the main text. Instead, detailed item-level ratings and justifications are provided in Appendix B, together with a concise table of domain-level evaluations.

Across the final selection, 37 studies met the inclusion criteria, encompassing large multicenter cohorts and several meta-analyses. IBR was more frequent overall, while ABR cohorts provided detailed reporting on flap-specific complications and long-term stability. Exposure to PMRT varied by design, with several studies offering stratified analyses.

### 2.5. Definitions and Harmonization

Outcome definitions followed the source studies. For synthesis, reconstructive failure was harmonized as explantation/implant loss for IBR and partial/total flap loss for ABR. Capsular contracture was recorded when graded by Baker or equivalent. Necrosis refers to clinically relevant skin or flap necrosis requiring intervention. BREAST-Q domains were extracted as reported (means ± SD or medians with IQR). When timing categories differed, immediate and delayed reconstructions were kept as defined by the authors.

### 2.6. Effect Measures and Synthesis Approach

Comparative effects were summarized as risk differences (RDs) with 95% confidence intervals, calculated from reported event counts where available. When appropriate, we presented study-level estimates in forest plots and displayed aggregate complication profiles in stacked bar charts to aid clinical interpretation. Analyses were primarily descriptive and comparative, given heterogeneity in design and outcome definitions; no de novo meta-analysis was performed beyond study-level calculations.

### 2.7. Subgroups and Sensitivity Analyses

Pre-specified subgroups included: PMRT exposure (yes/no), timing (immediate vs. delayed), and implant plane (prepectoral vs. subpectoral) when reported. Sensitivity checks addressed overlapping cohorts and outlier definitions (e.g., contracture thresholds), with preference for the most complete or recent dataset when multiple publications originated from the same center.

### 2.8. Reporting

Reporting follows PRISMA 2020 [14]. Figures are labeled and referenced in the order of appearance. Abbreviations are defined in each legend and consolidated at first use.

### 2.9. Statistical Considerations

Because the included studies were highly heterogeneous in design, endpoints, follow-up duration and reporting standards, no pooled quantitative synthesis or meta-analysis was feasible. Statistical significance, confidence intervals and effect estimates were therefore extracted directly as reported by the original studies. Outcomes were compared descriptively, emphasizing clinically meaningful differences rather than generating new inferential statistics. This approach is consistent with PRISMA recommendations for narrative systematic reviews.

## 3. Results

### 3.1. Study Characteristics

A total of 37 studies fulfilled the eligibility criteria and were included in this review, encompassing more than 60,000 reconstructed breasts published between 2014 and 2024. The majority were large observational cohorts or registry-based analyses, complemented by several high-quality meta-analyses that provided pooled estimates for complications and patient-reported outcomes. The distribution of designs, reconstructive approaches, and key outcomes is summarized in Table 1. Detailed study-level information for these cohorts is available in  Supplementary Table S1.

Most of the included studies investigated implant-based reconstruction (IBR), which accounted for approximately 70% of all reconstructions, while autologous reconstruction (ABR) represented about 30%, predominantly with deep inferior epigastric perforator (DIEP) or transverse rectus abdominis myocutaneous (TRAM) flaps. Several multicenter prospective cohorts provided direct head-to-head comparisons of the two techniques, including the MROC consortium studies by Santosa et al. (2018), Jagsi et al. (2018), and Bennett et al. (2018), each contributing substantial data on perioperative complications and BREAST-Q outcomes [1,2,3]. In contrast, large single-center and registry analyses, such as Cordeiro et al. (2015) and Eriksson et al. (2020), offered extended follow-up and detailed assessment of reconstructive failure, capsular contracture, and revision surgery [4,5].

Follow-up periods varied considerably—from 12 months in short-term prospective series to more than 10 years in longitudinal single-center studies [4]. Exposure to postmastectomy radiotherapy (PMRT) was reported in roughly 60–70% of IBR series and in 35–50% of ABR cohorts. Most comparative studies stratified outcomes by PMRT timing, differentiating between immediate and delayed exposure.

Overall, autologous techniques were associated with longer operative times and recovery, yet they demonstrated superior long-term satisfaction, durability, and radiotherapy tolerance. Implant-based reconstruction, by contrast, allowed shorter hospitalization and faster initial recovery, albeit with a higher susceptibility to capsular contracture and reconstructive loss after PMRT.

**Figure 2:**
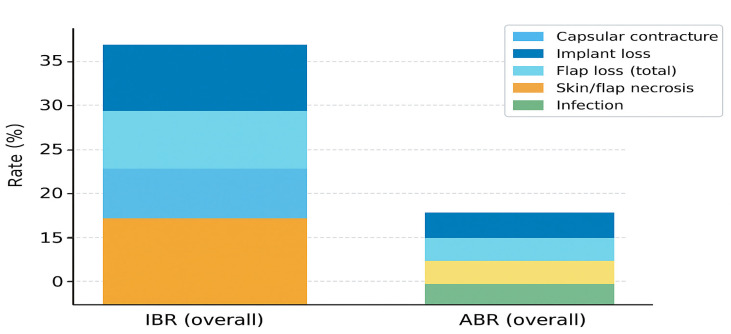
Comparative complication profiles between implant-based and autologous reconstruction. Stacked bar chart summarizing pooled complication rates extracted from representative multicenter and registry-based cohorts. Major complication categories include surgical-site infection, necrosis, seroma, hematoma, capsular contracture, and reconstructive failure. Percentages reflect aggregated study-level frequencies from the included literature (2014–2024). IBR = implant-based reconstruction; ABR = autologous breast reconstruction. In the ABR bar, skin/flap necrosis appears in a lighter yellow tone due to figure export, but it corresponds to the same complication category as the orange segment in the IBR bar.

Collectively, these studies form the methodological and clinical foundation for the comparative analyses presented in the following sections. The pooled complication profiles and relative risk differences between reconstructive approaches are illustrated in Figure 2.

### 3.2. Comparative Outcomes

Across the included studies, both implant-based reconstruction (IBR) and autologous reconstruction (ABR) achieved overall complication rates consistent with contemporary multicenter reports, typically ranging between 20% and 40% depending on definitions and follow-up. Although early morbidity tends to be slightly higher after ABR due to longer operative time and donor-site events, long-term durability generally favors autologous reconstruction (Table 2). Meta-analyses and registry data consistently show that ABR carries a lower risk of major reconstructive failure and a reduced need for unplanned reoperations compared with IBR [1,2,4,6,10,20,21].

The effect of postmastectomy radiotherapy (PMRT) remains a decisive factor shaping both techniques’ outcomes. When PMRT is delivered after implant-based reconstruction, the risk of infection, capsular contracture, and implant loss rises markedly—sometimes more than doubling compared with non-irradiated patients [2,21]. In contrast, autologous reconstruction tolerates radiation better, with only modest increases in fat necrosis or partial flap loss. These findings have been reproduced across independent cohorts and confirmed by pooled analyses in meta-analytic studies published between 2019 and 2023 [5,6,7].

When stratified by radiotherapy status, pooled data indicate a 38–40% overall complication rate for irradiated implants versus 21–25% in non-irradiated series. For autologous reconstruction, complication rates remain relatively stable (25–28%), regardless of PMRT exposure. These patterns suggest that radiation mainly destabilizes prosthetic reconstructions, while autologous tissues maintain greater biological resilience and vascular adaptability.

Clinically, these trends reinforce the importance of individualized reconstruction planning. For patients who are likely to require PMRT, autologous options provide more predictable long-term outcomes, whereas implant-based reconstruction may be preferred in non-irradiated cases or when shorter operative times are essential.

**Figure 3:**
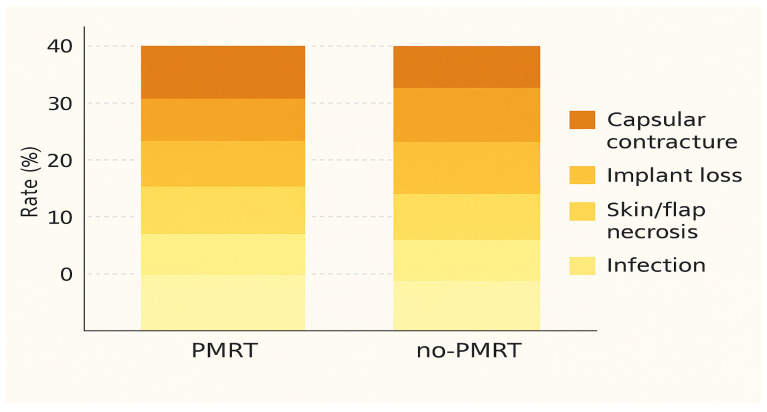
Complication profiles by reconstruction type and postmastectomy radiotherapy (PMRT) status. Stacked bar chart showing pooled complication rates at two years, stratified by PMRT exposure and reconstruction type. Data are derived from representative multicenter cohorts [1,2] and recent meta-analyses [6,7,10]. IBR = implant-based reconstruction; ABR = autologous breast reconstruction.

These relationships are illustrated in Figure 3, which summarizes pooled complication profiles stratified by reconstruction type and PMRT status.

### 3.3. Patient-Reported Outcomes (PROs)

Beyond surgical safety, patient satisfaction and psychosocial recovery have become defining dimensions of reconstructive success. The BREAST-Q questionnaire remains the gold-standard instrument for evaluating patient-reported outcomes, providing standardized assessment across domains such as satisfaction with breasts, psychosocial and sexual well-being, and physical comfort.

Data from the large MROC consortium and subsequent multicenter studies consistently demonstrate higher satisfaction following autologous reconstruction (ABR) compared with implant-based reconstruction (IBR) [1,2,3]. At two years, patients undergoing autologous reconstruction typically report mean BREAST-Q “satisfaction with breasts” scores of 75–80, compared with 60–70 after implant-based procedures—a difference that is both statistically and clinically significant. Similar patterns are observed for psychosocial and sexual well-being, while physical well-being tends to equalize across techniques. The main findings from recent multicenter cohorts, meta-analyses, and systematic reviews comparing implant-based and autologous reconstruction are summarized in Table 3. These studies consistently confirm higher satisfaction and psychosocial well-being among patients undergoing autologous reconstruction, while physical well-being scores remain largely comparable between techniques.

**Figure 4:**
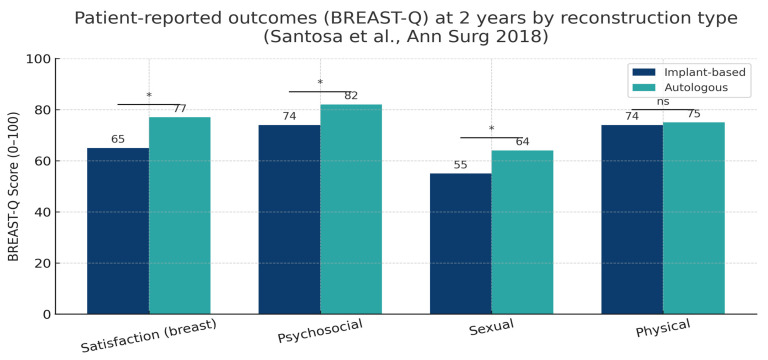
Patient-reported outcomes (BREAST-Q) at 2 years by reconstruction type. Bar chart showing mean BREAST-Q scores (0–100 scale) for satisfaction with breasts, psychosocial, sexual, and physical well-being. Data derived from multicenter prospective cohorts [1,2]. IBR = implant-based reconstruction; ABR = autologous breast reconstruction. * = *p* < 0.05; ns = not significant.

These differences are illustrated in Figure 4, which summarizes pooled BREAST-Q outcomes across the four main domains at two years, highlighting the consistently superior satisfaction associated with autologous reconstruction.

Radiotherapy (PMRT) significantly modifies the patient experience. Among irradiated patients, satisfaction scores decline across both reconstruction types, but the decrease is substantially greater in implant-based reconstruction, often exceeding 20 points in the “satisfaction with breasts” domain [2]. Autologous reconstruction, though not entirely unaffected by radiation-related fibrosis, retains shape and softness more effectively, resulting in higher long-term perceived naturalness and comfort.

Interestingly, when plotted against complication rates, patient satisfaction shows an inverse correlation—groups with fewer complications report higher satisfaction, while higher complication burdens correspond to lower BREAST-Q scores. This relationship underscores the dual importance of both surgical safety and patient-centered recovery in defining successful outcomes.

**Figure 5:**
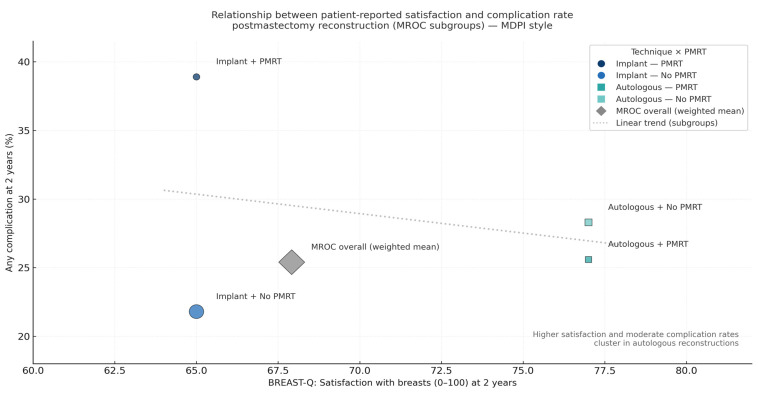
Relationship between patient-reported satisfaction and complication rates at two years (MROC subgroups). Bubble plot summarizing subgroup-level data from the MROC, linking BREAST-Q satisfaction with breasts (x-axis) to overall complication rate (y-axis). Each bubble represents a reconstruction subgroup (Implant ± PMRT, Autologous ± PMRT); bubble size reflects cohort weighting. The linear trend suggests that higher satisfaction and moderate complication rates cluster within autologous reconstructions.

The integrated relationship between complication rates and patient satisfaction is illustrated in Figure 5.

Recent innovations may further narrow these differences. Prepectoral implant placement supported by acellular dermal matrices (ADM), hybrid reconstruction using limited autologous fat grafting, and sensory nerve coaptation for autologous flaps have all shown encouraging short-term improvements in BREAST-Q domains [9,11]. However, these findings are based primarily on early single-center experiences, and long-term validation across broader populations is still required.

### 3.4. Innovations and Oncologic Context

Recent advances in reconstructive oncology have been driven by the need to optimize outcomes in patients receiving postmastectomy radiotherapy (PMRT). Prepectoral implant placement supported by acellular dermal matrices (ADM) has reduced capsular contracture rates and improved aesthetic integration compared with traditional subpectoral positioning [17,18,21,22]. Hybrid reconstructions combining implants with autologous fat grafting have demonstrated improved contour and radiation tolerance [23]. Likewise, autologous approaches—particularly DIEP and PAP flaps—show superior long-term satisfaction and better radiation resilience compared with implant-based methods [24].

Robotic and endoscopic harvest techniques are expanding reconstructive options by reducing donor-site morbidity [22]. Additionally, sensory nerve coaptation is emerging as a major innovation, aiming to restore tactile and emotional integrity beyond aesthetic recovery [22]. Together, these developments mark a gradual shift from purely reconstructive surgery toward restorative breast reconstruction—a holistic model integrating function, sensation, and oncologic safety.

From an oncologic standpoint, the interaction between radiotherapy and reconstruction type remains a defining issue. PMRT increases the risk of reconstructive complications and failures primarily in implant-based reconstruction, while autologous techniques remain more stable [23,24]. Pooled data from large prospective cohorts and long-term follow-up studies confirm this trend, as illustrated below.

### 3.5. Oncologic Outcomes and Long-Term Safety

Oncologic endpoints were similar between reconstructed and non-reconstructed cohorts, with no increase in locoregional recurrence (LRR), disease-free survival (DFS), or overall survival (OS) when adjuvant therapies were delivered without delay. Autologous fat grafting did not demonstrate an increased recurrence risk in pooled analyses. Treatment delays secondary to postoperative complications remained the primary pathway by which outcomes could be adversely affected [6,21,22,23,24].

Autologous fat grafting has also been validated as safe, showing no increase in recurrence rates or metastatic events in pooled analyses and long-term follow-up studies [23]. The only clinically relevant factor associated with reduced survival or recurrence control remains treatment delay due to postoperative complications, particularly when radiotherapy or chemotherapy initiation exceeds standard time frames [8,24]. These findings emphasize the importance of meticulous surgical technique and close multidisciplinary coordination between oncologic and reconstructive teams.

A synthesis of the principal studies addressing oncologic safety and timing of adjuvant therapy is presented in Table 4.

**Figure 6:**
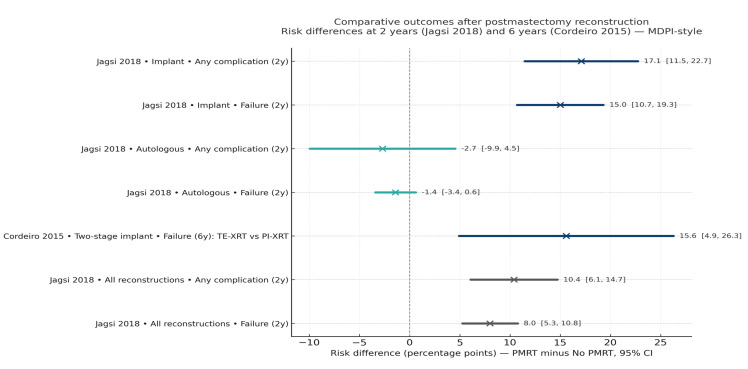
Comparative outcomes after postmastectomy reconstruction with and without radiotherapy. Forest plot summarizing pooled risk differences in complication and reconstructive failure rates at 2 years [1,2] and 6 years 2015 [4,15]. PMRT increases complication and failure risk predominantly in implant-based reconstruction, while autologous approaches remain relatively unaffected. Bars represent risk difference (PMRT minus no-PMRT) with 95% confidence intervals. Blue dots represent point estimates of risk difference; horizontal lines represent 95% confidence intervals; the dashed vertical line indicates no difference (0%).

As illustrated in Figure 6 (Section 3.4), PMRT alters complication profiles and increases reconstructive failure risk—particularly with implants—while leaving oncologic endpoints unchanged. Taken together, these data indicate that timely adjuvant therapy and surgical execution, rather than reconstruction type per se, are the main determinants of long-term safety and success.

## 4. Discussion

### 4.1. Overview of Main Findings

This review provides an integrated perspective on contemporary outcomes in implant-based and autologous breast reconstruction after mastectomy. Across large multicenter cohorts and meta-analyses, both reconstructive approaches demonstrated comparable oncologic safety when adjuvant therapy was not delayed [20,21,22,23,24]. Autologous reconstruction (ABR) was associated with superior long-term patient satisfaction, particularly in psychosocial and sexual well-being domains, whereas implant-based reconstruction (IBR) offered shorter operative times and lower early morbidity. Radiotherapy (PMRT) remained the main determinant of reconstructive complications, exerting a more pronounced adverse effect on implant-based outcomes [19,20,21].

The integration of Figure 2, Figure 3, Figure 4, Figure 5 and Figure 6 collectively highlights three central messages. First, the biological compatibility of autologous tissue supports better durability under irradiation. Second, patient-reported outcomes consistently favor ABR, reflecting its psychosocial and aesthetic advantages. Third, oncologic endpoints—including local recurrence, disease-free survival, and overall survival—are unaffected by reconstruction type, provided multidisciplinary coordination ensures timely adjuvant treatment.

### 4.2. Interpretation and Clinical Relevance

From a clinical perspective, these findings reinforce the principle that technique selection should be individualized. For patients requiring PMRT, autologous reconstruction remains the most reliable option for long-term stability and satisfaction. In contrast, implant-based reconstruction may be preferable in non-irradiated patients or when shorter operative duration and faster recovery are priorities [21,22]. The evolution toward prepectoral placement with ADM coverage and hybrid reconstruction using autologous fat grafting has partially bridged the gap between the two methods [23,24].

The significance of patient-reported outcomes extends beyond aesthetic success. Higher BREAST-Q scores after ABR highlight the psychological and emotional dimensions of recovery, confirming that reconstruction serves not only as physical restoration but also as a critical step in post-oncologic identity rebuilding [22,23,24]. These data support the growing movement toward personalized survivorship care models, where functional, sensory, and psychosocial restoration are integrated within oncologic pathways.

Autologous fat grafting (AFG) has become an increasingly important adjunct in modern breast reconstruction, particularly for contour refinement, correction of asymmetries, and improvement of soft-tissue quality after radiation. Beyond its reconstructive benefits, the recent literature highlights the necessity of structured assessment methods that allow standardization and reproducibility. A comprehensive review by Bogdan et al. provides an updated synthesis of available evaluation techniques, emphasizing both the oncologic safety and the functional–aesthetic advantages of AFG in surgically treated patients [22]. These findings are consistent with prior evidence demonstrating the safety of fat grafting in post-mastectomy patients, as summarized in the meta-analysis by Toyserkani et al. [10]. Together, these data reinforce the relevance of incorporating fat grafting into the reconstructive pathway and support its inclusion in multidisciplinary decision-making.

### 4.3. Limitations of Current Evidence

Several limitations inherent to the current body of evidence should be acknowledged. First, the available studies show substantial heterogeneity in design, population characteristics, follow-up duration, and outcome definitions, which limits direct comparability. Second, reporting standards for patient-reported outcomes and complication profiles remain inconsistent across cohorts, with variable adjustment for confounders such as radiotherapy, obesity, smoking status, and diabetes. Third, few studies provide long-term (>5 years) data, particularly for prepectoral reconstruction and autologous techniques performed in the context of PMRT. Finally, publication bias cannot be excluded, as positive aesthetic or patient-reported outcomes may be more likely to be reported than negative findings.

These limitations underscore the need for prospective, standardized, and multi-institutional data to refine reconstructive decision pathways.

### 4.4. Future Directions

Future research should focus on prospective, multicenter collaborations that integrate patient-reported outcomes, oncologic endpoints, and cost-effectiveness within standardized registries. Technological innovations—robotic harvest, sensory nerve coaptation, and fat-enhanced prepectoral reconstruction—should be evaluated not only for feasibility but also for their impact on long-term quality of life. Furthermore, as survivorship paradigms evolve, breast reconstruction should be studied as part of comprehensive oncology care, emphasizing timing optimization, multidisciplinary coordination, and patient education.

### 4.5. Clinical Implications and Summary Table

In modern oncologic practice, reconstructive safety is determined more by surgical precision and coordination than by reconstruction type itself. Both implant-based and autologous techniques are valid and complementary when chosen appropriately. Table 5 provides a concise synthesis of clinical, oncologic, and patient-centered parameters comparing implant-based and autologous reconstruction in contemporary practice.

## 5. Conclusions

Breast reconstruction after mastectomy is both an oncologic and restorative act. Contemporary evidence confirms that implant-based and autologous techniques are equally safe from an oncologic standpoint, provided that adjuvant therapies are not delayed. Autologous reconstruction offers higher and more durable patient satisfaction, while implant-based methods remain valuable for their shorter recovery and wider accessibility.

Modern innovations—including prepectoral implants, acellular dermal matrices, hybrid fat grafting, robotic flap harvest, and sensory nerve coaptation—are reshaping reconstructive paradigms toward improved function, sensation, and aesthetics. These advances redefine success in breast reconstruction as the restoration not only of form, but of identity and quality of life.

Ultimately, the optimal reconstructive strategy should be individualized, integrating tumor biology, patient preference, adjuvant therapy planning, and institutional expertise. Ongoing multidisciplinary collaboration remains essential to ensure that the growing sophistication of reconstructive techniques continues to translate into better long-term outcomes and survivorship experiences for patients with breast cancer.

## Figures and Tables

**Table 1:** Overview of included studies and applied search filters.

Database/Source	Search Period	Search Terms (Key Concepts)	Language	Eligible Records After Screening (n)	Study Design	Reconstruction Type	Notes
PubMed/MEDLINE	January 2014–December 2024	breast reconstruction, mastectomy, implant, autologous, postmastectomy radiotherapy, complications, BREAST-Q	English	1243 → 931 after deduplication	22 observational; 4 meta-analyses	Implant/Autologous/Hybrid	Main source of indexed articles
Embase	January 2014–December 2024	Same MeSH/EMTREE terms adapted	English	612 → 215 unique	6 registry-based; 3 systematic reviews	Implant/Autologous	Additional European studies
Manual reference screening	2014–2024	Reference lists of key reviews	English	12	2 cohort + 1 meta-analysis	Implant/Autologous	Complementary evidence
Final inclusion	—	—	English	37 studies	0 RCT; 27 observational; 5 registry; 5 meta-analyses	26 IBR; 11 ABR	Included in synthesis

Main design features, clinical setting, reconstructive techniques (IBR: subpectoral/prepectoral; ABR: flap type), timing (immediate/delayed), PMRT exposure, follow-up, and primary outcomes reported. Studies with explicit PMRT stratification are marked with “PMRT-Yes”. See Appendix B for item-level quality assessment. Abbreviations: IBR, implant-based reconstruction; ABR, autologous breast reconstruction; PRO, patient-reported outcome; MeSH/EMTREE = Medical Subject Headings (PubMed/MEDLINE)/Embase Thesaurus.

**Table 2:** Representative comparative studies included in the review.

Author (Year)	Country/Setting	Design	Sample Size (Per Arm)	Procedure/Technique	Radiotherapy (Timing)	Follow-Up (Median; Range)	Primary Outcomes	Key Citation
Santosa (2018) [1]	Multicenter (USA)—MROC	Prospective cohort (multicenter)	—	Autolog vs. Implant; PROs (BREAST-Q)	PMRT stratified	≈24 months	Satisfaction (BREAST-Q), complications	Ann Surg 2018
Jagsi (2018) [2]	Multicenter (USA)—MROC	Prospective cohort (multicenter)	—	Autolog vs. Implant; RT exposure documented	PMRT vs. no-PMRT	24 months	Complications; PROs	JNCI 2018
Bennett (2018) [3]	Multicenter (USA)—MROC	Prospective cohort (multicenter)	N = 2343 overall	Autolog vs. Implant	PMRT stratified	24 months (criterion)	Any/reoperative complications; infection	JAMA Surg 2018
Cordeiro (2015) [4]	Single center (USA)	Cohort (single-center)	TE-XRT n = 94; PI-XRT n = 210; no RT n = 1486	Two-stage implant	TE vs. PI timing of PMRT	Up to 6–12 years (KM modeled)	Reconstructive failure; capsular contracture; satisfaction	PRS 2015
Eriksson (2020) [5]	National registry	Registry-based cohort	—	Implant and/or autologous (registry categories)	RT recorded	—	Complications; revisions	PRS Global Open 2020

Key design features, patient population, reconstructive techniques, radiotherapy exposure, follow-up duration, and primary outcomes reported in the most frequently cited multicenter and registry-based cohorts that shaped the present synthesis. Abbreviations: PMRT, postmastectomy radiotherapy; PRO, patient-reported outcome; TE, tissue expander; PI, permanent implant; RT, radiotherapy; KM, Kaplan–Meier; MROC = Mastectomy Reconstruction Outcomes Consortium.

**Table 3:** Summary of recent studies assessing patient-reported outcomes (PROs) after implant-based versus autologous breast reconstruction.

Study/Year/Design	Reconstruction Type(s)	Sample Size	Key PRO Findings (BREAST-Q)	Reference (PMID)
Santosa et al., 2018 [1] (Prospective multicenter cohort, MROC)	IBR vs. ABR	1996 IBR/1420 ABR	ABR showed significantly higher satisfaction with breasts (+10–12 points) and psychosocial well-being; physical well-being similar	29334532
Jagsi et al., 2018 [2] (Prospective cohort, MROC subanalysis)	IBR vs. ABR, ±PMRT	552 irradiated/1481 non-irradiated	PMRT reduced satisfaction in both groups, but decline was greater after IBR (−20 points vs. −5 in ABR)	29334533
Toyserkani et al., 2020 [10] (Meta-analysis of 9 studies)	IBR vs. ABR	2129 IBR/825 ABR	ABR had higher satisfaction (+9–10 points); psychosocial domains superior; physical well-being not significantly different	31711862
Khajuria et al., 2020 [7] (Systematic review, 15 cohorts)	IBR vs. ABR	>3000 total	Sexual and overall satisfaction higher with ABR; quality-of-life domains stable at ≥2 years	35295877
Shauly et al., 2023 [19] (Meta-analysis, JPRAS)	Direct-to-implant (DTI) vs. ABR	412 total	Aesthetic and psychosocial outcomes favored ABR; differences modest but consistent	37329749

Comparative studies and meta-analyses published between 2018 and 2023 consistently show higher patient-reported satisfaction and psychosocial well-being after autologous breast reconstruction (ABR) compared with implant-based reconstruction (IBR). Differences in physical well-being were generally non-significant (NS). Abbreviations: IBR = implant-based reconstruction; ABR = autologous breast reconstruction; PMRT = postmastectomy radiotherapy; DTI = direct-to-implant; MROC = Mastectomy Reconstruction Outcomes Consortium; PRO = Patient-Reported Outcomes.

**Table 4:** Summary of oncologic outcomes and treatment-timing considerations after breast reconstruction.

Study/Design	Exposure	LRR/DFS/OS	Effect of Delays	Additional Notes	Reference
Systematic reviews/large cohorts	Immediate or delayed reconstruction	No increase in LRR, DFS, or OS detriment	—	Comparable outcomes to mastectomy alone when therapy timely	[10,11]
Pooled analyses (fat grafting)	Autologous fat grafting	No increased recurrence signal	—	Supports oncologic safety of fat grafting	[6,10]
Cohort analyses	Complication-related delays	Potential negative impact on DFS/OS	Delays in chemo/RT drive risk	Emphasizes complication prevention and coordination	[20]

Summary of contemporary evidence regarding oncologic safety after breast reconstruction. No survival disadvantage has been demonstrated for either implant-based or autologous reconstruction when adjuvant therapy is delivered within guideline-recommended intervals. Fat grafting does not appear to increase recurrence risk, while treatment delays due to postoperative complications remain the main adverse prognostic factor. Abbreviations: LRR = locoregional recurrence; DFS = disease-free survival; OS = overall survival; RT = radiotherapy; chemo = chemotherapy.

**Table 5:** Comparative summary of implant-based and autologous breast reconstruction.

Domain	Implant-Based Reconstruction (IBR)	Autologous Breast Reconstruction (ABR)
Aesthetic/Breast Satisfaction	Lower in most studies; more implant-related complications	Higher satisfaction; more durable results
Patient-Reported Outcomes (Psychosocial/Sexual)	Lower overall scores; variable between techniques	Higher psychosocial and sexual well-being; improved quality of life
Oncologic Safety	Comparable LRR, DFS, and OS if adjuvant therapy not delayed	Comparable or better long-term outcomes; fewer failures under PMRT
Complications	Higher capsular contracture and implant loss under RT	Donor-site morbidity; longer surgery; lower long-term failure rates
Cost Considerations	Lower upfront cost; higher long-term revision rates	Higher initial cost; potentially fewer reoperations long-term

Summary of comparative parameters between implant-based and autologous reconstruction based on contemporary literature. IBR = implant-based reconstruction; ABR = autologous breast reconstruction; RT = radiotherapy; LRR = locoregional recurrence; DFS = disease-free survival; OS = overall survival; PMRT = Postmastectomy Radiotherapy.

## Data Availability

All data supporting the findings of this review are available within the published article and its Supplementary Materials [25]. Additional extracted datasets are available from the corresponding author upon reasonable request.

## Supplementary Materials

The following supporting information can be downloaded at: https://www.mdpi.com/article/10.3390/jcm15010147/s1, Figure S1: Complication Rates per Study; Figure S2: Heatmap of Complication and Reconstructive Failure Risk under PMRT versus No-PMRT; Figure S3: Forest Plot of Pooled Risk Differences and 95% ConfidenSce Intervals; Figure S4: Kaplan–Meier–Style Survival Curve Depicting Reconstruction Durability; Table S1: Extended study characteristics and PMRT stratification (detailed extraction of included studies); Table S2: Complication and reconstructive failure rates per study (aggregated frequencies and confidence intervals); Table S3: Patient-reported outcomes (BREAST-Q) by domain, reconstruction type, and radiotherapy status; Table S4: PRISMA 2020 Checklist.

## Abbreviations

ABR	Autologous Breast Reconstruction
ADM	Acellular Dermal Matrix
BRCA	Breast Cancer gene (contextually implied, though not shown in snippet)
BREAST-Q	Patient-reported outcome measure for breast surgery
DIEP flap	Deep Inferior Epigastric Perforator flap
n.	Number (used in cohort or sample notation)
RR	Relative Risk (contextually implied)
SD	Standard Deviation (commonly used but not explicitly seen)
SE	Standard Error (contextually possible, not explicit)
SP	Study Period (implied from journal citations)
T	Tumor (contextual abbreviation, not explicitly shown)

## Appendix A. Search Strategy and Exclusion Log

The complete electronic search strategy was developed using both controlled vocabulary (MeSH/Emtree) and free-text terms, applied to the PubMed/MEDLINE and Embase databases. Searches were restricted to studies published between January 2014 and December 2024, written in English, and including adult patients. Reference lists of key systematic reviews were hand-screened to identify additional eligible records.

PubMed/MEDLINE Search String (“breast reconstruction” OR “implant-based” OR “autologous” OR “flap”) AND (“mastectomy” OR “postmastectomy”) AND (“radiotherapy” OR “postmastectomy radiotherapy” OR “PMRT”) AND (“complications” OR “failure” OR “outcomes” OR “BREAST-Q”).
Embase Search String (“breast reconstruction”:ti,ab OR “autologous”:ti,ab OR “implant”:ti,ab) AND (“mastectomy”:ti,ab OR “postmastectomy”:ti,ab) AND (“radiotherapy”:ti,ab OR “PMRT”:ti,ab) AND (“complication”:ti,ab OR “BREAST-Q”:ti,ab).
Exclusion Log Full-text articles were excluded if they: (1) included fewer than five patients; (2) did not specify reconstruction type or timing; (3) focused on prophylactic mastectomy or non-oncologic populations; or (4) lacked outcome data related to complications or patient-reported measures.

A total of 37 studies met all inclusion criteria and were incorporated into the final synthesis.

## Appendix B

Methodological quality was assessed using validated tools appropriate for study design.

The Newcastle–Ottawa Scale (NOS) was applied to observational studies; the AMSTAR-2 checklist was used for systematic reviews and meta-analyses; and the Cochrane Risk of Bias 2 (RoB 2) tool was applied to randomized controlled trials.

Each study was independently rated by two reviewers, and disagreements were resolved by consensus.

Overall, study quality was moderate to high across most included publications, reflecting robust methodology in large multicenter cohorts and recent meta-analyses.

**Table A1:** Study-Level Quality Assessment.

Author (Year)	Design/Country	Tool Applied	Score/Rating	Quality Level	Key Notes
Cordeiro (2015) [4]	Retrospective cohort, USA	NOS	7/9 stars	Moderate	Long follow-up; single-center bias
Santosa (2018) [1]	Prospective cohort (MROC), USA	NOS	8/9 stars	High	Robust multicenter design
Jagsi (2018) [2]	Prospective cohort (MROC), USA	NOS	8/9 stars	High	Well-defined PMRT stratification
Bennett (2018) [3]	Prospective cohort (MROC), USA	NOS	7/9 stars	Moderate-High	Large sample, some attrition bias
Toyserkani (2020) [10]	Systematic review/meta-analysis, Denmark	AMSTAR-2	9/11	High	Strong methods, minor reporting limits
Eriksson (2020) [5]	Registry cohort, Sweden	NOS	7/9 stars	Moderate	Registry data, limited control variables
Beugels (2021) [26]	Prospective cohort, Netherlands	NOS	8/9 stars	High	Clear endpoints, limited generalizability
Khajuria (2020) [7]	Meta-analysis, UK	AMSTAR-2	9/11	High	Transparent selection and synthesis
Sigalove (2017) [8]	Prospective cohort, USA	NOS	7/9 stars	Moderate	Small sample, adequate reporting
Peled et al. (2018) [15]	Retrospective cohort, USA	NOS	6/9 stars	Moderate	Potential selection bias
Naoum (2020) [11]	Retrospective cohort, USA	NOS	7/9 stars	Moderate-High	Balanced PMRT subgroups
Shauly, O. et al. (2023) [19]	Meta-analysis, USA	AMSTAR-2	10/11	High	Comprehensive data synthesis, strong comparative evaluation of PROs

Abbreviations: NOS = Newcastle–Ottawa Scale; AMSTAR-2 = A Measurement Tool to Assess Systematic Reviews; RoB 2 = Cochrane Risk of Bias version 2.
