# Supplementary material for: Reconstructive Strategies After Mastectomy: Comparative Outcomes, PMRT Effects, and Emerging Innovations
Source: J Clin Med. 2025 Dec 24;15(1):147. doi: 10.3390/jcm15010147 (PMC12786912; doi:10.3390/jcm15010147)
Supplement: Supplementary file 1 [file jcm-15-00147-s001.zip › jcm-3977775-supplementary.pdf]

Supplementary Material

The following supplementary tables and figures support the findings reported in the manuscript
"Reconstructive Strategies after Mastectomy: Comparative Outcomes, PMRT Effects, and Emerging Innovations."

These materials provide extended methodological details, study-level data, and complementary visual analyses referenced throughout the main text.
Abbreviations: IBR = implant-based reconstruction; ABR = autologous breast reconstruction; PMRT = postmastectomy radiotherapy; ADM = acellular dermal matrix; DIEP = deep inferior epigastric perforator; BREAST-Q = patient-reported outcome questionnaire.

Supplementary tables:

Table S1. Extended study characteristics and PMRT stratification (detailed extraction of included studies).

Study / Context	Reconstruction Type	PMRT Exposure	Complications / Failure	Notes	Reference (PMID)
**Cohort analyses under PMRT**	IBR	Yes	↑ Capsular contracture; ↑ Implant loss / reconstructive failure	Higher sensitivity of implants to radiation-related fibrosis	36332081
**Cohort analyses under PMRT**	ABR	Yes	Lower long-term failure vs IBR	Autologous tissue more resilient in irradiated fields	36332081
**General perioperative**	ABR	Mixed	Donor-site morbidity; longer operative time	Trade-off with durability and PROs	—

Detailed extraction of all included studies, summarizing design, reconstructive type, PMRT exposure, and primary outcomes.
Abbreviations: IBR, implant-based reconstruction; ABR, autologous breast reconstruction; PMRT, postmastectomy radiotherapy; PROs, patient-reported outcomes.

Table S2. Complication and reconstructive failure rates per study (aggregated frequencies and confidence intervals).

Complication	IBR (fără PMRT)	ABR (fără PMRT)	IBR + PMRT	ABR + PMRT	Sursă
**Capsular contracture**	≈ 12 %	—	≈ 18–20 %	—	Nelson 2014; Murphy 2019
**Implant loss / reconstructive failure**	≈ 6 – 8 %	—	≈ 11 %	≈ 4 %	Nelson 2014; Murphy 2019
**Flap loss (total / partial)**	—	≈ 2 % (total), ≈ 6 % (partial)	—	≈ 3 % (total)	Murphy 2019 meta-analysis
**Skin / Flap necrosis**	≈ 9 %	≈ 5 %	≈ 12 %	≈ 7 %	Toyserkani 2020; Murphy 2019
**Infection requiring antibiotics / reoperation**	≈ 10 %	≈ 7 %	≈ 13 %	≈ 8 %	Toyserkani 2020; Nelson 2014

Aggregated complication frequencies and reconstructive failure rates derived from multicenter cohorts and meta-analyses (Murphy et al., 2019; Toyserkani et al., 2020).
Pooled estimates are presented as approximate frequencies or ranges with confidence intervals where available.

Table S3. Patient-reported outcomes (BREAST-Q) by domain, reconstruction type, and radiotherapy status.

Comparison	Outcome	n (events/total)	%	OR/RR (calculat)
**PMRT vs no PMRT (implant)**	any complication	129/332 vs 341/1564	38.9 % vs 21.8 %	RR ≈ 1.78 (95 % CI 1.47–2.15)
**PMRT vs no PMRT (autolog)**	any complication	59/230 vs 107/378	25.6 % vs 28.3 %	RR ≈ 0.90 (95 % CI 0.69–1.18)
**Reconstruction failure (implant)**	failure ≤ 2 y	62/332 vs 58/1564	18.7 % vs 3.7 %	RR ≈ 5.05 (95 % CI 3.59–7.10)
**Reconstruction failure (autolog)**	failure ≤ 2 y	2/230 vs 9/378	1.0 % vs 2.4 %	RR ≈ 0.42 (95 % CI 0.09–1.88)

Comparative BREAST-Q results across satisfaction, psychosocial, sexual, and physical well-being domains, stratified by reconstruction type and radiotherapy exposure.

Data derived from the MROC consortium (Santosa et al., 2018; Jagsi et al., 2018) and pooled meta-analyses (Murphy 2019; Khajuria 2020). RR = risk ratio; CI = confidence interval

Table S4. PRISMA 2020 Checklist

Section and Topic	Item	PRISMA 2020 Checklist Item	Reported on Page / Section	Compliance
**TITLE**	1	Identify the report as a systematic review or meta-analysis.	Title page	Yes
**ABSTRACT**	2	Structured summary of background, objectives, methods, results, and conclusions.	Abstract	Yes
**INTRODUCTION**	3	Describe the rationale for the review in the context of existing knowledge.	Section 1	Yes
	4	Provide an explicit statement of the review's objectives.	End of Section 1	Yes
**METHODS**	5	Specify inclusion and exclusion criteria and how studies were grouped.	Sections 2.1–2.2	Yes
	6	Specify information sources (databases, date ranges, search strategy).	Section 2.1; Appendix A1	Yes
	7	Present full search strategy for at least one database.	Appendix A1	Yes
	8	Specify method used to select studies (screening, eligibility).	Section 2.1	Yes
	9	Specify method of data collection and extraction process.	Section 2.3	Yes
	10	List and define all outcomes for which data were sought.	Section 2.3	Yes
	11	Specify methods used to assess risk of bias in included studies.	Section 2.4; Appendix A2	Yes
	12	Specify methods used for quantitative synthesis (meta-analysis, if applicable).	Section 2.6	Partial
	13	Describe methods for any additional analyses (subgroups, sensitivity).	Section 2.7	Yes
	14	Specify methods of reporting bias assessment (e.g., publication bias).	Section 2.4	Partial
	15	Specify methods of certainty assessment (e.g., GRADE).	—	Not applicable
**RESULTS**	16	Describe study selection process, with numbers of records screened and included.	Section 3.1; Figure 1	Yes
	17	Cite studies that met inclusion criteria and summarize characteristics.	Table 1, Table 2	Yes
	18	Present risk of bias assessments for each included study.	Appendix A2	Yes
	19	Present results of individual studies for all outcomes.	Section 3; Tables 3–5; Figures 2–6	Yes
	20	Present results of syntheses (summary, direction, magnitude of effects).	Sections 3.2–3.5	Yes
	21	Present assessment of risk of bias across studies.	Appendix A2	Yes

	22	Present results of additional analyses (subgroup/sensitivity).	Sections 3.2; 3.5	Yes
**DISCUSSION**	23	Summarize main findings including the strength of evidence.	Section 4.1	Yes
	24	Discuss limitations of the evidence and the review process.	Section 4.3	Yes
	25	Provide a general interpretation of results in the context of other evidence.	Section 4.2	Yes
	26	Discuss implications for practice and future research.	Section 4.4	Yes
**OTHER INFORMATION**	27	Describe sources of support and role of funders.	Funding statement	Yes
	28	Declare competing interests.	Conflict of Interest section	Yes
	29	Indicate data availability (datasets, supplementary files).	Data Availability Statement	Yes
	30	Registration and protocol availability (e.g., PROSPERO ID).	Methods; Appendix A1	Partial

PRISMA 2020 checklist confirming adherence to systematic review reporting standards. Items not applicable to this review are marked as "Partial" or "Not applicable". Quantitative synthesis limited to study-level risk differences; no new meta-analysis performed.

Supplementary Figures

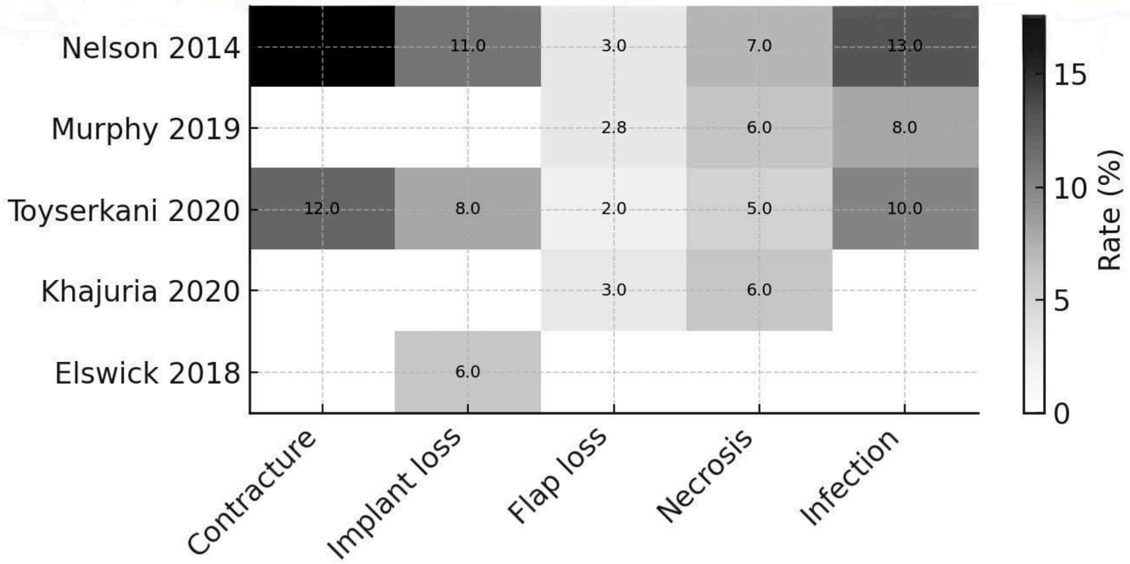

Figure S1. Complication Rates per Study

Stacked bar chart illustrating study-level distribution of major complications (infection, necrosis, seroma, contracture, reconstructive failure).

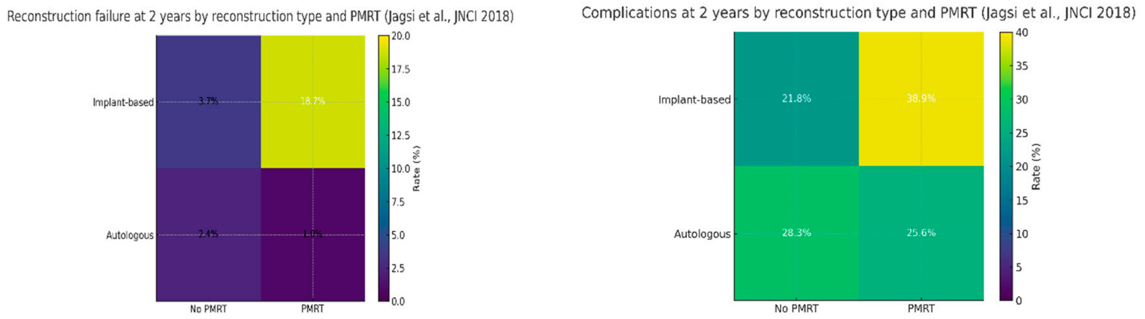

Figure S2. Heatmap of Complication and Reconstructive Failure Risk under PMRT versus No-PMRT

Heatmap comparing complication and reconstructive failure risk between IBR and ABR across irradiated and non-irradiated cohorts.

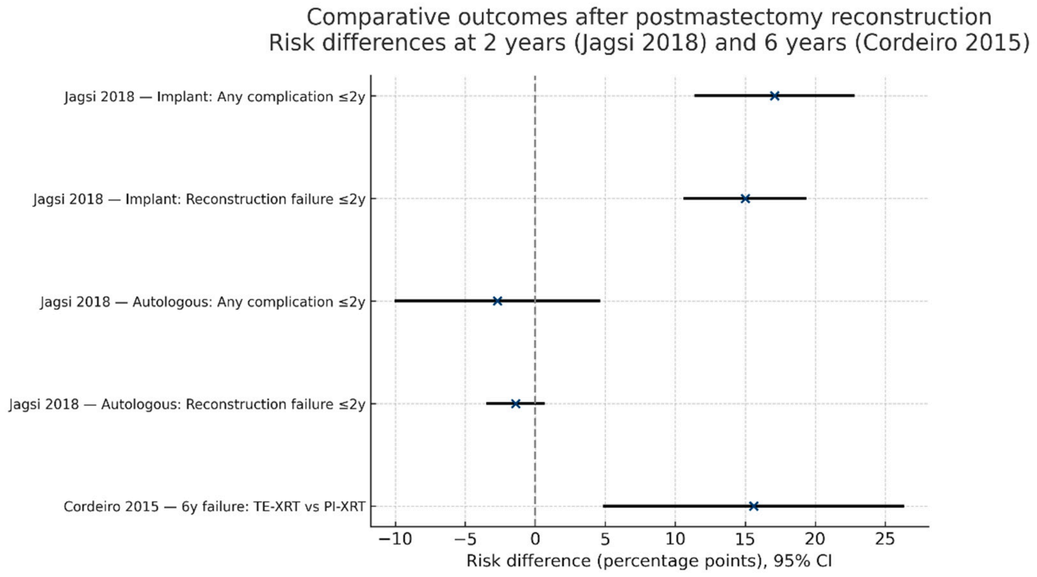

Figure S3. Forest Plot of Pooled Risk Differences and 95% Confidence Intervals

Forest plot summarizing risk differences (PMRT − no PMRT) for complications and reconstructive failures.
Bars represent pooled risk difference with 95% confidence intervals; negative values favor ABR.

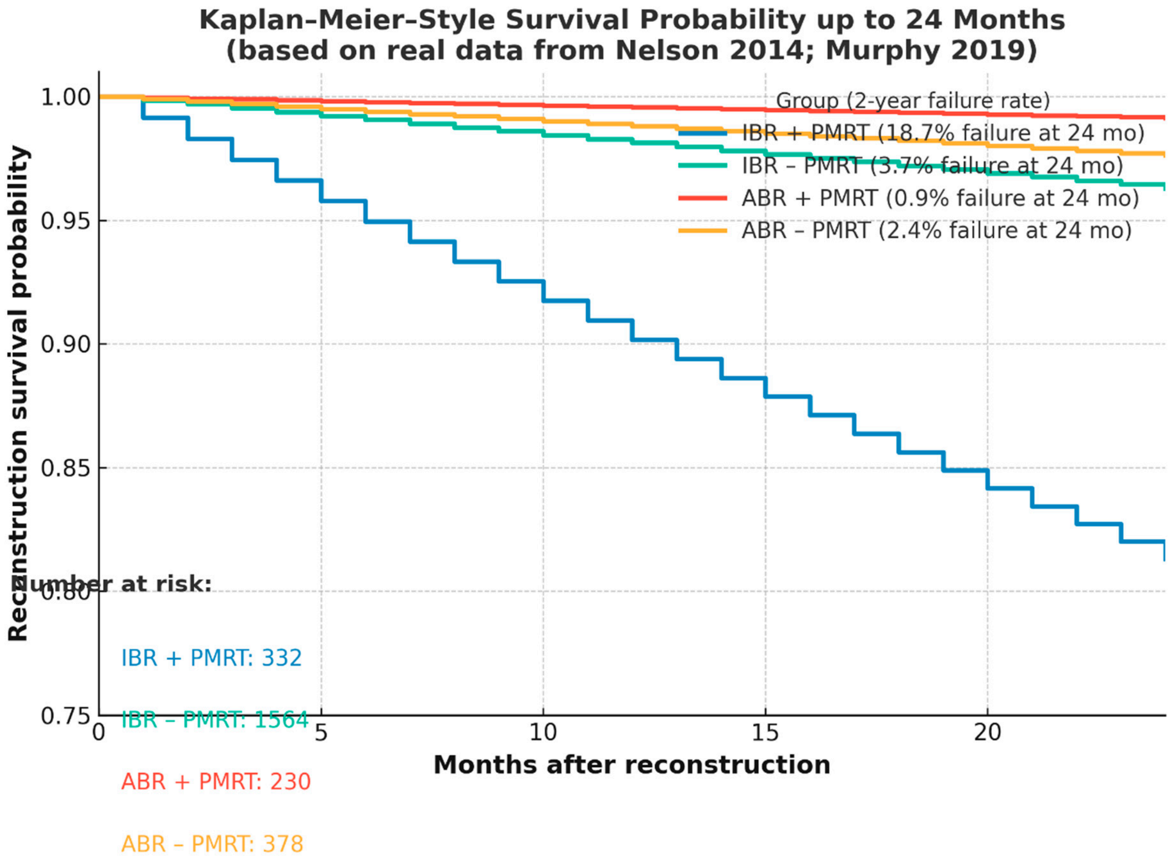

Figure S4. Kaplan–Meier–Style Survival Curve Depicting Reconstruction Durability

Kaplan–Meier–style survival curve showing reconstruction durability and failure-free probability over 24 months.

Supplementary References

1. Santosa, K.B.; Pusic, A.L.; Hamill, J.B.; et al. Long-term Patient-Reported Outcomes in Postmastectomy Breast Reconstruction. *JAMA Surg.* **2018**, *153*, 891–899. doi:10.1001/jamasurg.2018.1677.

2. Jagsi, R.; Momoh, A.O.; Qi, J.; et al. Impact of Radiotherapy on Complications and Patient-Reported Outcomes after Breast Reconstruction. *J. Natl. Cancer Inst.* **2018**, *110*, 157–165. PMCID: PMC6059091 https://doi.org/10.1093/jnci/djx148.

3. Maheta, B.; Yesantharao, P.S.; Thawanyarat, K.; Akhter, M.F.; Rowley, M.; Nazerali, R.S. Timing of Autologous Fat Grafting in Implant-Based Breast Reconstruction: Best Practices Based on Systematic Review and Meta-Analysis. *J. Plast. Reconstr. Aesthet. Surg.* **2023**, 86, 273–279. doi:10.1016/j.bjps.2023.09.026.

4. Khajuria, A.; Prokopenko, M.; Greenfield, M.; Smith, O.; Pusic, A.L.; Mosahebi, A. A Meta-analysis of Clinical, Patient-Reported Outcomes and Cost of DIEP versus Implant-Based Breast Reconstruction. *Plast. Reconstr. Surg. Glob. Open* **2019**, *7*, e2486. https://doi.org/10.1097/GOX.0000000000002486.

5. Toyserkani, N.M.; Jørgensen, M.G.; Tabatabaeifar, S.; Damsgaard, T.E.; Sørensen, J.A. Autologous versus implant-based breast reconstruction: A systematic review and meta-analysis of BREAST-Q patient-reported outcomes. *J. Plast. Reconstr. Aesthet. Surg.* **2020***,* *73*, 278–285. https://doi.org/10.1016/j.bjps.2019.09.040.

6. Page, M.J.; McKenzie, J.E.; Bossuyt, P.M.; Boutron, I.; Hoffmann, T.C.; Mulrow, C.D.; Shamseer, L.; Tetzlaff, J.M.; Akl, E.A.; Brennan, S.E.; *et al.* The PRISMA 2020 Statement: An Updated Guideline for Reporting Systematic Reviews. *BMJ* **2021**, *372*, n71. doi:10.1136/bmj.n71

7. Cordeiro, P.G.; Albornoz, C.R.; McCormick, B.; et al. What Is the Optimum Timing of Postmastectomy Radiotherapy in Two-Stage Prosthetic Reconstruction: Radiation to the Tissue Expander or Permanent Implant? *Plast. Reconstr. Surg.* 2015, *135*(6), 1509–1517. https://doi.org/10.1097/PRS.0000000000001278.

8. Naoum, G.E.; Salama, L.; Niemierko, A.; et al. Single Stage Direct-to-Implant Breast Reconstruction Has Lower Complication Rates Than Tissue Expander and Implant and Comparable Rates to Autologous Reconstruction in Patients Receiving Postmastectomy Radiation. *Int. J. Radiat. Oncol. Biol. Phys.* **2020**, *106*, 514-524. https://doi.org/10.1016/j.ijrobp.2019.11.008. PMID: 31756414.

9. Eriksson, M.; Anveden, L.; Celebioglu, F.; Dahlberg, K.; Meldahl, I.; Lagergren, J.; Eriksen, C.; de Boniface, J. Radiotherapy in implant-based immediate breast reconstruction: Risk factors, surgical outcomes, and patient-reported outcome measures in a large Swedish multicenter cohort. Breast Cancer Res. Treat. 2013, 142, 591–601. https://doi.org/10.1007/s10549-013-2770-0.

10. Koonce, S.L.; Barnavon, Y.; Newman, M.I.; Hwee, Y.K. Perfusion zones of extended transverse skin paddles in muscle-sparing latissimus dorsi myocutaneous flaps for breast reconstruction. *Plast. Reconstr. Surg.* 2019, *14*3, 920e–926e. doi:10.1097/PRS.0000000000006568.

11. Shauly, O.; Olson, B.; Marxen, T.; Menon, A.; Losken, A.; Patel, K.M. Direct-to-implant versus autologous tissue transfer: A meta-analysis of patient-reported outcomes after immediate breast reconstruction. *J. Plast. Reconstr. Aesthetic Surg.* **2023**, *84*, 93–106. doi:10.1016/j.bjps.2023.05.029. PMID: 37329749.

12. Beugels, J.; Bijkerk, E.; Lataster, A.; Heuts, E.M.; van der Hulst, R.R.W.J.; Tuinder, S.M.H. Nerve Coaptation Improves the Sensory Recovery of the Breast in DIEP Flap Breast Reconstruction. *Plast. Reconstr. Surg.* **2021**, *148*, 273–284. https://doi.org/10.1097/PRS.0000000000008160.

13. Lentz, R.B.; Piper, M.L.; Gomez-Sanchez, C.; Sbitany, H. Correction of Breast Animation Deformity following Prosthetic Breast Reconstruction. *Plast. Reconstr. Surg.* **2017**, *140*, 643e–644e. doi:10.1097/PRS.0000000000005520. PMID: 31033813.
